# Supplementary material for: The genome sequence of the Grey Shoulder-knot, Lithophane ornitopus (Hufnagel, 1766)
Source: Wellcome Open Res. 2024 Apr 23;9:214. [Version 1] doi: 10.12688/wellcomeopenres.21280.1 (PMC11632219; doi:10.12688/wellcomeopenres.21280.1)
Supplement: Peer review history

# Reviewer report: Reviewer response for version 1

**DOI:** 10.21956/wellcomeopenres.23536.r99431

**Published:** 2024-12-10

**Authors:** Vinaya Shetty

**Affiliations:** 1 Department of Entomology, Texas A&M University, College Station, Texas, USA

The authors describe the genome sequencing, assembly, and annotation of *Lithophane ornitopus* (grey shoulder knot moth) as part of the Darwin Tree of Life project. High molecular weight DNA and RNA were extracted using standardized protocols, followed by sequencing on PacBio HiFi and Illumina NovaSeq platforms. The genome was assembled using Hifiasm, scaffolded with Hi-C data, and evaluated for quality and completeness with tools like BUSCO and Merqury. The study highlights a comprehensive workflow for generating high-quality genomic resources while adhering to ethical and legal standards for sample collection and data sharing.

**Comments and suggestions are as below.**

**Abstract**

- The assembly method (e.g., long-read, short-read sequencing, hybrid assembly) is not mentioned. Including this would provide insights into the methodology and quality of the assembly.
- Key quality metrics for genome assembly, such as N50 values, BUSCO scores, or levels of completeness, are not mentioned. These metrics are essential for evaluating the reliability and usability of the assembly.
- Briefly mention whether the gene count aligns with expectations or if any notable patterns were identified.

**Background**

 The description of the moth’s forewings and etymology is interesting but somewhat lengthy. This could be condensed to focus more on its ecological significance and evolutionary context, which would better justify the importance of assembling its genome. For example, why is *Lithophane ornitopus* an important species for genomic study? Are there broader implications for understanding Lepidoptera biodiversity or adaptation?

**Specific comments**

- Condense descriptive details about morphology and focus more on ecological and evolutionary importance.
- Highlight the scientific motivation for the genome assembly in the context of Lepidoptera or broader biodiversity research.
- Discuss potential applications of this genome in addressing taxonomic or ecological questions.

**Genome Sequence Report**

- While the specimen details are included (e.g., location, body parts used for sequencing), the report could benefit from a brief mention of why a male specimen was chosen, as sex-specific differences in genomic content (e.g., ZW sex chromosomes) may be relevant.
- The report lacks a comparative analysis with genome assemblies of other moth species or closely related taxa. Including such comparisons would provide context for the significance of the assembly metrics and highlight any unique features of *Lithophane ornitopus*.
- The report notes that contigs corresponding to the second haplotype are deposited separately, but it does not discuss the potential biological implications of this duplication or how it might affect downstream analyses.

**Methods**

- The dissection of the thorax and use of cryogenic disruption are mentioned, but there is no justification for selecting thorax tissue for DNA extraction and abdomen tissue for RNA extraction. Including a brief rationale would improve clarity.
- While sequencing platforms and library preparation kits are listed, details such as read length, sequencing depth, and coverage for both DNA and RNA libraries are missing. These metrics are crucial for evaluating data adequacy for downstream applications.
- The manuscript mentions the use of BUSCO and Merqury for assembly evaluation but does not report any metrics (e.g., completeness scores, QV scores). A summary of these results would significantly enhance the section.
- While Table 3 lists tools and versions, it would be helpful to align these versions explicitly with the methods described. For example, specifying which tool versions were used for Hi-C analysis or mitochondrial genome assembly ensures precision.

Are sufficient details of methods and materials provided to allow replication by others?

Partly

Is the rationale for creating the dataset(s) clearly described?

Yes

Are the datasets clearly presented in a useable and accessible format?

Yes

Are the protocols appropriate and is the work technically sound?

Yes

Reviewer Expertise:

Genetics and genomics

I confirm that I have read this submission and believe that I have an appropriate level of expertise to confirm that it is of an acceptable scientific standard, however I have significant reservations, as outlined above.

# Reviewer report: Reviewer response for version 1

**DOI:** 10.21956/wellcomeopenres.23536.r105631

**Published:** 2024-11-12

**Authors:** Eduardo Zattara, Lican Martinez

**Affiliations:** 1 CONICET-Universidad Nacional del Comahue, Instituto de Investigaciones en Biodiversidad y Medioambiente (Ringgold ID: 215197), San Carlos de Bariloche, Río Negro, Argentina; 2 Department of Invertebrate Zoology, National Museum of Natural History, Smithsonian Institution, Washington, DC, USA; 3 CONICET-Universidad Nacional del Comahue, Bariloche, Argentina

In this data note, Boyes and Zolli detail the assembly process for the genome of the noctuiid moth *Lithophane ornitus*, the Grey Shoulder-knot. They start with a nice introduction to the sequenced organism, and then report the main features of the resulting assembled and annotated genome. The methods used represent the standard methods used for DTOL projects, and yield what is likely to be a chromosomal-level assembly (although a karyotype for the species would help assess this).

 While some steps in the final curation of the assembly were manual and thus harder to document and reproduce independently by a third party, this is unlikely to affect the usefulness of this resource.

Are sufficient details of methods and materials provided to allow replication by others?

Partly

Is the rationale for creating the dataset(s) clearly described?

Yes

Are the datasets clearly presented in a useable and accessible format?

Yes

Are the protocols appropriate and is the work technically sound?

Yes

Reviewer Expertise:

Evo-devo, genomics, transcriptomics, regeneration, conservation

We confirm that we have read this submission and believe that we have an appropriate level of expertise to confirm that it is of an acceptable scientific standard.

# Reviewer report: Reviewer response for version 1

**DOI:** 10.21956/wellcomeopenres.23536.r99423

**Published:** 2024-09-26

**Authors:** Will Nash

**Affiliations:** 1 Earlham Institute, Norwich, England, UK

The authors present a chromosome level genome assembly for the Noctuid moth, the Grey Shoulder Knot (Lithophane ornitopus), sequended as part of the Darwin Tree of Life Project. The assembly is 508.6Mb, has an N50 of 17.2Mb, and represents 99% of the lepidoptera_odb10 BUSCO set. The genome has been sequenced and assembled with cutting edge methods. 

 The Background is well written and informative. It would be useful to reference the known occurence data for this species: https://www.gbif.org/species/1759668

 The Genome Sequencing Report shows the assembly to be highly accurate (QV 69.5) and well representative of the chromosome level (99% of the assembly in chromosomes). 18,397 protein coding genes are also annotated. 

 The methods represent a state-of-the-art approach to single insect genome sequencing and assembly.

 I am not satisfied that the methods are fully reproducible as the Hi-C protocol is under documented.

Are sufficient details of methods and materials provided to allow replication by others?

Partly

Is the rationale for creating the dataset(s) clearly described?

Yes

Are the datasets clearly presented in a useable and accessible format?

Yes

Are the protocols appropriate and is the work technically sound?

Yes

Reviewer Expertise:

Landscape Genomics, Bioinformatics, Genome Assembly

I confirm that I have read this submission and believe that I have an appropriate level of expertise to confirm that it is of an acceptable scientific standard, however I have significant reservations, as outlined above.
